# Ozone therapy: Mechanisms and clinical applications - A review

**DOI:** 10.6026/973206300213369

**Published:** 2025-09-30

**Authors:** Marshalein Anthony, Satyalakshmi Komarraju, Sathyanath D., Shrikanth Muralidharan

**Affiliations:** 1 Department of Ozone, National Institute of Naturopathy, Ministry of Ayush, Pune, India; 2 Department of Naturopathy and Yoga, National Institute of Naturopathy, Ministry of Ayush, Pune, India; 3 Department of Natural Therapeutics, National Institute of Naturopathy, Ministry of Ayush, Pune, India; 4 Department of Research, National Institute of Naturopathy, Ministry of Ayush, Pune, India

**Keywords:** Ozone therapy, oxidative stress, immune modulation

## Abstract

Ozone therapy is an evolving medical modality within naturopathy, integrative medicine and regenerative healthcare. Therefore, it is of interest to review synthesizes historical development, biochemical properties, mechanisms of action, administration methods and clinical applications. Thus, the need for multicentred randomized controlled trials, exploration of molecular mechanisms including epigenetic effects and integration with regenerative medicine is highlighted.

## Background:

A developing field in naturopathy, ozone treatment focusses primarily in integrative and regenerative medicine and is characterised by the therapeutic application of ozone gas (O_3_), a triatomic molecule with strong oxidative capabilities. Accurate oxygen-fed ozone generators are used to create modern medical ozone, which is a calibrated O_2_-O_3_ gas mixture that can be administered via parenteral, topical and insufflation techniques [1, 2]. By activating the nuclear factor erythroid 2-related factor 2 (Nrf2) signalling pathway, this triggers a series of adaptive reactions in cellular redox systems. Under oxidative stress, Nrf2 moves to the nucleus and increases the activity of antioxidant enzymes such as glutathione peroxidase (GPx), heme-oxygenase-1 (HO-1) and superoxide dismutase (SOD), strengthening cellular defences and repair processes [3, 4]. Pro-inflammatory cytokines (such as IL-1β and TNF-α) are first released and then transforming growth factor-beta (TGF-β) and IL-10 are released in a compensatory anti-inflammatory response [5, 6]. Clinical research has investigated ozone therapy for a variety of ailments, such as peripheral artery disease, musculoskeletal diseases, chronic viral infections and even oncological supportive care [7, 8]. Moreover, ozone therapy may potentially improve microcirculation and oxygen delivery in ischemic tissues because of its capacity to boost tissue oxygenation through enhanced erythrocyte synthesis of 2, 3-diphosphoglycerate (2, 3-DPG) [9]. Ozone therapy is approved and widely practiced in countries like Germany, Italy and Cuba [10]. Therefore, it is of interest to report the clinical applications of ozone in healthcare practices.

## Historical background of ozone therapy:

## Discovery and early observations (19th Century):

German-Swiss chemist Christian Friedrich Schönbein of the University of Basel made the initial identification of ozone (O_3_) in 1839. Schönbein discovered a unique scent during electrolysis and electric current flow through water tests. This smell was eventually determined to be ozone, which comes from the Greek word ozein, which means "to smell" [11]. Using cold discharge techniques, physicist Werner von Siemens created the first usable ozone generator by 1857 [12].

## Bactericidal and antiseptic use (Late 19th - Early 20th Century):

The first widespread application of ozone in medicine was in 1915, during World War I, when German doctors used it for wound care, gangrene prevention and localised infection management [13, 14]. Dr. C. Lender proposed in 1881 that ozone may be used to sterilise operating rooms and surgical tools. Reports on the effective use of ozonated water and gas to treat chronic wounds and tuberculous illnesses were then published in the Lancet in 1902 [15].

## Development of medical ozone generators (1930s-1950s):

When German engineer Erwin Payr and doctor H.A. Fisch introduced medically certified ozone generators in the 1930s, it was a turning point. In Europe around this time, ozone therapy was used in dermatology, gynaecology and general surgery, especially in Germany, Austria and Switzerland [16]. Ozone therapy's professional credibility in Central Europe was cemented in 1958 with the founding of the German Medical Society for Ozone Therapy (Deutsche Gesellschaft für Ozontherapie) [17].

## Scientific rationalization and biochemical insights (1960s-1980s):

Dr. Hans Wolff developed Major Autohemotherapy (MAH) in the 1960s, which involves drawing blood, ozonating it and then injecting it again. This approach of systemic ozone treatment became commonplace. Biochemical research into the mechanism of ozone was conducted in the 1970s and 1980s, particularly in the Soviet Union and Cuba. The application of ozonated oils and rectal insufflation in the treatment of ischaemic disorders and chronic diseases has been thoroughly investigated by Cuban researchers under the direction of Dr. Silvia Menéndez [18]. Italian physiology professor Dr. Velio Bocci postulated that ozone stimulates antioxidant mechanisms, immunological regulation and oxygen metabolism through regulated oxidative stress [19].

## Regulation, expansion and modern use (1990s-Present):

Ozone therapy was institutionalised in a number of nations starting in the 1990s. Its effectiveness in some indications, including lumbar disc herniation, diabetic foot ulcers and chronic wounds, was first supported by a number of randomised controlled trials (RCTs) and meta-analyses in the 2000s and 2010s [20, 21]. More insight into the immunological and oxidative mechanisms of ozone has been gained in recent years thanks to molecular-level studies of Nrf2 (nuclear erythroid factor 2) activation, mitochondrial biogenesis and cytokine regulation.

## Chemistry and properties of ozone:

## Molecular structure and stability:

Ozone has a molecular geometry with an O-O-O bond angle of approximately 116.8°, consistent with sp^2^ hybridization of the central oxygen atom. The molecule exists in a resonance hybrid state, distributing the double bond character equally between both O-O bonds, making it relatively unstable compared to diatomic oxygen (O_2_) [22]. Ozone's high-energy configuration makes it highly unstable under physiological conditions, decomposing rapidly into molecular oxygen (O_2_) and a free oxygen atom (O°), which reacts readily with surrounding biomolecules.

## Generation of medical ozone:

## Two primary methods are used:

## Corona discharge method:

Oxygen is passed through a high-voltage electrical field. Results in partial dissociation of O_2_ molecules into atomic oxygen, which then recombines to form O_3_

## Ultraviolet (UV) radiation method:

UV radiation from the Sun splits oxygen molecules (O_2_) into individual oxygen atoms (O). These atoms then combine with other O_2_ molecules to form ozone (O_3_). Key substances that contribute to ozone depletion are chlorine and bromine compounds.

## Solubility and diffusion:

Ozone may pass through cell membranes and interact with proteins, phospholipid bilayers and plasma antioxidants because it is highly soluble in lipids and moderately soluble in water [2]. At 20-25 °C in ambient conditions, ozone's half-life is approximately 20-30 minutes. In biological fluids, its half-life is only milliseconds. Rapid decomposition makes real-time generation essential in clinical settings. Medical ozone is always administered as a freshly generated O_2_-O_3_ mixture, often in 95-99% O_2_ and 1-5% O_3_. The following flow chart (Figure 1 - see PDF) explains the Biochemical Effects of Ozone.

## Mechanism of action:

Immune modulation, redox signalling, microcirculation enhancement, mitochondrial bioenergetics, neuroendocrine regulation and antibacterial activity are some of the systems that are involved in these interactions [23].

## Oxidative preconditioning and redox signaling:

When ozone is added to biological fluids (blood, plasma and interstitial fluids), it quickly interacts with water, lipids and antioxidants to produce LOPs (like 4-hydroxynonenal and malondialdehyde) and ROS (like hydrogen peroxide [H2O_2_]) [1]. Through oxidative preconditioning brought on by these fleeting oxidative signals, the cell's antioxidant capacity is increased via nuclear factor erythroid 2-related factor 2 (Nrf2) signalling activation antioxidant enzymes such as glutathione peroxidase, catalase and superoxide dismutase (SOD) are upregulated. Redox equilibrium restoration in situations involving chronic oxidative stress (such as diabetes and ischaemia) [24].

## Modulation of the innate and adaptive immune system:

The following figure 2 (see PDF) shows the modulation of immunity by ozone [24, 25].

## Increase in tissue oxygenation:

By increasing erythrocyte glycolysis, improving O_2_ delivery (Bohr Effect), restoring erythrocyte membrane fluidity through decreased lipid peroxidation and increased Na+/K+ ATPase activity and lowering blood viscosity and improving erythrocyte deformability, ozone enhances tissue oxygenation [26].

## Anti-inflammatory mechanisms:

## Ozone therapy downregulates chronic inflammation through:

Inhibition of NF-κB translocation, promotion of pro-resolving cytokines such asIL-10, TGF-β and regulation of eicosanoid metabolism (↓ PGE_2_ synthesis) [5]. The Figure 3 (see PDF) highlights the antimicrobial effect of ozone.

## Neuromodulator and pain control:

Ozone therapy achieves analgesia by bringing about Reduction in substance P and bradykinin at nociceptive terminals and enhancement of endogenous opioid peptides (e.g., β-endorphins) [6].

## Methods of administration:

Below is a detailed classification and explanation of all primary and secondary methods of medical ozone application (Figure 4 - see PDF).

## Minor autohemotherapy (mAHT):

mAHT serves as an "autologous vaccine-like" immunologic intervention. It is particularly effective in allergic, viral and autoimmune conditions due to its antigenic presentation effects and immunologic priming [27]. The following Figure 5 (see PDF) shows the clinical application of ozone therapy [28, 29, 30-31].

## Contraindications:

Ozone therapy is contraindicated in certain conditions due to potential safety risks. These include first-trimester pregnancy, glucose-6-phosphate dehydrogenase (G6PD) deficiency, hyperthyroidism, severe anemia and active bleeding disorders.

## Future perspectives:

The future of ozone therapy lies in advancing research and technology. Key directions include conducting multi-center randomized controlled trials (RCTs) to strengthen clinical evidence, exploring ozone's epigenetic impacts for deeper mechanistic understanding, developing nanoparticle-bound ozone delivery systems for targeted and controlled release and integrating ozone therapy into regenerative medicine to enhance tissue repair and healing outcomes.

## Funding

Nil
